# Extracts from Proceedings of the Forest City Society of Dental Surgeons, Cleveland, Ohio, June 6th, 1871

**Published:** 1871-09

**Authors:** Henri L. Ambler


### EXTRACTS FROM PROCEEDINGS OF THE FOREST CITY SOCIETY OF DENTAL SURGEONS, CLEVELAND, OHIO, JUNE 6th, 1871.

The society was called to order by the President, Dr. C. R. Butler. The election of officers for the ensuing year resulted as follows:

*President*—C. R. Butler.
*Vice President*—B. T. Spelman.
*Recording Secretary*—H. L. Ambler.
*Corresponding Secretary*—C. Buffett.
*Treasurer*—L. Buffett.
*Councilmen*—Drs. Palmer, Harroun, Slosson.

The President remarked that he felt much honored, and was under great obligations to the society for a re-election, hoping that he might ever prove faithful to his charge. This society is a united and brotherly one in every sense of the word, and it is good for us that such is the case; and even as a profession, we are acknowledged to be more social and fraternal than our medical brothers. . In conversation, a dentist should not be confined entirely to professional matters, but let him take time to gain a general scientific knowledge; thus fitting himself, to fill in a creditable manner, any position which he may be called upon to occupy.

We should teach patients that ours is a science, an art, and a profession—not a mere trade. Ten years ago, a physician seldom, if ever, thought of calling a dentist for consultation with him; but now they are often found consulting together, and we regard it as only just and proper to give each his deserved position. Be on such good terms with your brothers, that should you have a difficult case, you feel at perfect liberty to call one of them to consult with you. Such a proceeding gives a patient greater confidence, they think you are really trying to do them good; thus may you help show them, that the diseases of the teeth and mouth are not of such trivial importance, that any tyro may treat them successfully. We may congratulate ourselves, that in our specialty, such great progress has been made during the last few years, and that a more thorough education of men for the profession is demanded and recognized by the people, as well as by those of our profession, who are ever ready to elevate and maintain a high standard in practice. Now, brethren, however much has been done in the past, we should not forget that much is required of us in the future; therefore, let us ever be zealous in well doing, that it may be said of us our part in the great work was well and faithfully performed.

Dr. Slosson remarked that those about to enter the profession, should have a good general education upon all scientific subjects, and be able to converse knowingly upon them, and especially should we aim to instruct the mass of people.

H. L. Ambler remarked that he had published in the *Ohio Farmer*, a weekly paper, a series of articles on "Preservation of the Teeth," believing this to be one of the best ways to educate the public, and bring them to a high appreciation of our science. Thinks that the main reason why more articles in regard to our profession, have not appeared in public papers, is because the publishers wanted pay, not deeming such matter of general interest enough to warrant them in putting it into their columns. However, such ideas, are fast becoming dissipated by the progress of dental surgery. Has also for the last three years, produced before the State Institute of Homœopathy, essays upon the importance of our specialty, and its close alliance to the practice of medicine. His last production, in the way of an artificial nose, was the restoration with pyroxiline, of the portion corresponding to the right ala.

Dr. Harroun related a case of diseased lower jaw, with a fistulous opening under the chin, caused by the growing together and ulceration of two roots, which by exostosis became firmly imbeded in the substance of the maxilla. Also said that he was still making a specialty of producing artificial obturators, and desires brotherly influence in sending him cases. Thus far, he has never seen a case of inflammation resulting from such fixtures made of rubber.

Dr. Butler described a case where all the superior teeth were removed, and the patient had been wearing an artificial denture for years. There had been a discharge of pus from the anterior alveolar ridge for some months. A "capital" operation had been advised by examining surgeons, but finally, he administered an anæsthetic, made an incision and inserted a probe, striking a hard body, which was at once diagnosed as a tooth; being easily removed with a pair of small forceps, it was found to be a cuspid, which was imbedded horizontally in the alveolar arch. The tooth was of good fair size, with the enamel partially denuded.

Dr. Butler described four difficult cases of irregularity, remarking that you should use as simple fixtures as possible, and do not be anxious to move the teeth *too* fast. Bands of pyroxiline are preferable to any metallic substance, as you can shape it quickly to any case; the color is good, and there is just spring enough to give the teeth a moderate pull, such as you desire. Rubber rings and silk ligatures are used to place around the teeth for tension; the rings should be made wide to prevent their slipping under the gum, as narrow ones are liable to do; the rubber helps separate the teeth, and gives them something of a chance to come into a good position. Often puts in a stay plate to assist the band; sometimes drilling holes in it, putting in boxwood pins in order to get pressure on certain teeth. All cases must be left entirely to the operator's care, giving patients to particularly understand, that should they have any trouble, to come immediately to him. Always make definite engagements, so that you may be able to note the progress of the case, and do not talk to them as if it was of little importance whether they attended faithfully to it or not.

Irregularity is hereditary; for we have seen the same deformity of the teeth, in sister, aunt and cousin; but we believe it can generally be overcome, by carefully regulating for generations.

Dr. Harroun recommends Watt & Williams' metal for stay plates.

Dr. Spelman makes beautiful models, by the use of soapstone on his "plaster Paris." In pivoting teeth, he covers the end of the root with gold, in order to obtain a good joint.

Dr. Palmer exhibited, and showed the application of four beautiful filling instruments of his own manufacture; they are particularly for reaching parts of a tooth most difficult to fill successfully with the use of the mallet. He refers to the posterior lingual cusps, of inferior bicuspids and molars; these are points most difficult to condense the gold laterally and carry it forward, for we should have lateral condensation as we progress in building up lost crowns; also in filling where a good portion of the crown remains, it will be found that here are difficult points to carry the gold directly to the walls. He is in favor of modeling fillings near as possible, to the desired contour, as you proceed, thus saving much time and strength in finishing. The style of instruments, and their adaptation, called out many praise-worthy remarks.

Dr. Butler remarked that thick foil, in his hands, would produce a better edge, and lie up to the thin wall of a tooth, much better than thin foil; nor does it require any more force in condensing. With thick foil you can make a stronger contour filling, and get a better surface with less labor. When you use "coffer dam" you have perfect dryness, and can put in the gold with smooth points if desirable, for they tend to spread or stretch the foil up to the walls. Uses a burnisher considerably in filling or restoring. Fills root canals with os-artificial and fibres of gold as a carrying vehicle, considers it the quickest and best method.

Dr. Spelman read a report on mechanical dentistry, which says, that at present the laboratories of many of our best men are closed, and a great portion of the balance are in a fruitless condition. Those to whom we looked, expecting them to bring out something new and valuable in this department, have left it in sorrow and disgust. It is true that flasks and vulcanizers have been improved, but this adds nothing to the pleasure and comfort of our patients. The time necessary to vulcanize a set of teeth, has been lessened, by adding more sulphur to the rubber, still we fail to see how this makes the rubber stronger or more lasting, or how it makes it less poisonous. The color has been made to please the eye by the addition of mercury, and at the expense of strength. We think there is a desire on the part of patients and the profession, to return to the honored gold and continuous gum work. Since it has been discovered by the more honest of the profession, that rubber is such a miserable thing for dental use, a great effort has been made to discover something else as cheap, and less obnoxious. Thus we have Aluminum, Rose Pearl, Adamantine, Weston's metal, Watt & Williams', and many others, which are superior to rubber; where they undergo no oxidation, they do not act deleteriously upon the general health, or local tissue of the mouth; they are conductors of heat, which is much in their favor. These metals do not contain mercury or sulphur, and can be made thinner, stronger, and less clumsy than rubber; they can also be repaired without injury. Here follows an *extended* compilation of the views of many of our leading men, as expressed in journals and associations, the general opinion being adverse to the use of rubber.

From the American Dental Association: "silver has passed away, and is now almost forgotten. Rubber, too, will soon be no more. The hand writing on the wall 'bespeaks its doom, and predicts for it a reputation for evil which will long curse its memory.'"

Your committee think that the absorption and loss of hard tissue, is the greatest where rubber plates are worn, which probably is owing to the mercury or non-conductile property of the material.

Adjourned, after a pleasant session of two days.

HENRI L. AMBLER, *Rec. Sec.*

